# Iron Uptake Mechanisms in Marine Phytoplankton

**DOI:** 10.3389/fmicb.2020.566691

**Published:** 2020-11-05

**Authors:** Robert Sutak, Jean-Michel Camadro, Emmanuel Lesuisse

**Affiliations:** ^1^Department of Parasitology, Faculty of Science, Charles University, BIOCEV, Vestec, Czechia; ^2^CNRS, Institut Jacques Monod, Université de Paris, Paris, France

**Keywords:** iron, phytoplankton, iron uptake, micro-algae, ocean

## Abstract

Oceanic phytoplankton species have highly efficient mechanisms of iron acquisition, as they can take up iron from environments in which it is present at subnanomolar concentrations. In eukaryotes, three main models were proposed for iron transport into the cells by first studying the kinetics of iron uptake in different algal species and then, more recently, by using modern biological techniques on the model diatom *Phaeodactylum tricornutum*. In the first model, the rate of uptake is dependent on the concentration of unchelated Fe species, and is thus limited thermodynamically. Iron is transported by endocytosis after carbonate-dependent binding of Fe(III)’ (inorganic soluble ferric species) to phytotransferrin at the cell surface. In this strategy the cells are able to take up iron from very low iron concentration. In an alternative model, kinetically limited for iron acquisition, the extracellular reduction of all iron species (including Fe’) is a prerequisite for iron acquisition. This strategy allows the cells to take up iron from a great variety of ferric species. In a third model, hydroxamate siderophores can be transported by endocytosis (dependent on ISIP1) after binding to the FBP1 protein, and iron is released from the siderophores by FRE2-dependent reduction. In prokaryotes, one mechanism of iron uptake is based on the use of siderophores excreted by the cells. Iron-loaded siderophores are transported across the cell outer membrane via a TonB-dependent transporter (TBDT), and are then transported into the cells by an ABC transporter. Open ocean cyanobacteria do not excrete siderophores but can probably use siderophores produced by other organisms. In an alternative model, inorganic ferric species are transported through the outer membrane by TBDT or by porins, and are taken up by the ABC transporter system FutABC. Alternatively, ferric iron of the periplasmic space can be reduced by the alternative respiratory terminal oxidase (ARTO) and the ferrous ions can be transported by divalent metal transporters (FeoB or ZIP). After reoxidation, iron can be taken up by the high-affinity permease Ftr1.

## Introduction

Iron is vital for almost all forms of life, with aerobic organisms having particularly large requirements for this element. Iron is abundant in the respiratory and photosynthetic electron transport chains, in the form of heme and iron-sulfur prosthetic groups, and it is also present in the iron-rich nitrogenase protein complex of diazotrophs (Buren et al., 2020). Iron was abundant in its reduced form, Fe^2+^, in the Proterozoic ocean (Falkowski, 2006). However, the levels of bioavailable iron have decreased considerably over time, with the increase in oxygen levels in the environment, because the solubility of the oxidized form, Fe^3+^, is extremely low in neutral and basic conditions. Marine organisms have, therefore, had to adapt, to maintain sufficiently high levels of this increasingly scarce resource. Low iron availability nevertheless limits phytoplankton growth in about 30–50% of the ocean, in the vast HNLC (high-nutrient low-chlorophyll) regions, in eastern boundary upwelling regions and at the deep chlorophyll maximum (Hogle et al., 2018). The existence of this iron limitation has been demonstrated in experiments in which the addition of iron to HNLC waters has been shown to boost the growth of phytoplankton considerably, generating spectacular blooms (Boyd et al., 2007). The bioavailability of iron (as Fe’ released from acidic ligands) to phytoplankton may decrease still further in the future, due to the acidification of the ocean as atmospheric CO_2_ levels increase, while the bioavailability of iron from oxy-hydroxide colloids should not change significantly (Shi et al., 2010), and the release of iron upon dust deposition may be enhanced (Li et al., 2017).

Most of the iron in the ocean is complexed with organic ligands, the nature of which remains unclear. Nevertheless, there is growing evidence to suggest that iron is associated with both small, well-defined ligands including siderophores, and with diverse macromolecular complexes (Gledhill and Buck, 2012; Shaked et al., 2020). Unchelated iron is present in hydrolyzed forms, ${\text{Fe}(\text{OH})}_{x}^{\left(3-x\right)+}$, the neutral tri-hydroxy species, Fe(OH)_3_, having an extremely low solubility. It has been shown that this pool of unchelated iron is the preferred source of iron for marine micro-algae (Lis et al., 2015b). However, given the very particular nature of the ocean environment (low iron concentration with episodic inputs, heterogeneous pool of iron complexes), it seems likely that highly novel mechanisms of iron uptake await our discovery in phytoplanktonic algae and bacteria. Dark/light cycles may also be involved in regulating iron uptake systems in phytoplankton species, because the photoreductive dissociation of natural ferric chelates or ferric colloids in seawater may increase the concentration of free iron (Fe^3+^aq) available for transport by more than two orders of magnitude (Sunda, 2001).

Terrestrial microorganisms and plants have two main iron uptake strategies, both of which have been characterized in the yeast *Saccharomyces cerevisiae* (for a review, see Sutak et al., 2008). In the first strategy, the various ferric complexes present in the environment are dissociated by reduction at the cell surface, and free ferrous iron is then taken up by the cells (Lesuisse and Labbe, 1989; Kosman, 2003). In the second strategy, siderophores excreted by the cells or produced by other bacterial or fungal species capture iron in the environment and are then taken up via specific high-affinity receptors (for a review, see Philpott, 2006). Much less is known about the strategies by which marine phytoplankton species acquire iron.

Many marine bacteria are known to produce siderophores (Butler, 1998, 2005), and it has been suggested that the marine diatoms *Thalassiosira pseudonana* (Armbrust et al., 2004) and *Phaeodactylum tricornutum* (Kustka et al., 2007) have a yeast-like reducing system. Several marine micro-algae have no ferric reductase activity and do not excrete siderophores (Sutak et al., 2012). These organisms have to cope with extremely low, subnanomolar concentrations of iron. The affinity constants of the known terrestrial iron uptake systems are in the micromolar range and would, therefore be inefficient in a marine environment. The iron acquisition strategies of marine phytoplankton must have evolved to cope with iron scarcity and the very particular conditions prevailing in the marine environment, which has a transition metal composition very different from that of terrestrial environments (Butler, 1998). Some progress has been made recently in our understanding of the mechanisms phytoplankton employs to cope with low iron levels in the marine environment, and the mechanisms involved often diverge from terrestrial models.

## Eukaryotes

### Different Models of Iron Uptake

**FIGURE 1:**
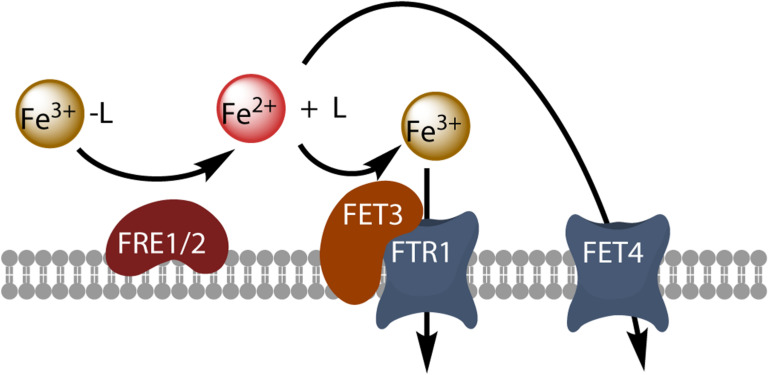
Model of reductive iron uptake in yeast. Ferric chelates (Fe^3+^-L) are dissociated by reduction at the cell surface, and free ferrous ions are taken up by the high-affinity Fet3-Ftr1 complex or by the divalent metal transporter Fet4.

Oceanic eukaryotic phytoplankton species have highly efficient mechanisms of iron acquisition, as they can take up iron from environments in which it is present at subnanomolar concentrations. By comparing the rate of iron uptake from free inorganic soluble ferric species (Fe(III)’) in several iron-limited phytoplankton species, Lis et al. (2015b) revealed a strong correlation of uptake rate constants (iron uptake rate/substrate concentration) between all the species tested, and showed that these uptake rate constants were proportional to surface area, suggesting that they had reached a universal upper limit (Lis et al., 2015b). Iron uptake may have been pushed up toward the maximum limits imposed by diffusion and ligand exchange kinetics (Sunda and Huntsman, 1995). Several authors have tried to identify the best iron sources for uptake in phytoplankton. The ocean contains a number of different physicochemical fractions of dissolved iron, including Fe(II), colloidal and inorganic Fe, and organically complexed iron. It is widely thought that organic iron-binding ligands complex more than 99% of the dissolved iron in the ocean (Gledhill and Buck, 2012; Boiteau et al., 2016). These organic ligands include siderophores, larger macromolecular complexes, such as humic compounds or exopolymeric substances, and ligands that remain to be identified (Gledhill and Buck, 2012). Even though most of the iron present is chelated, it has been suggested that unchelated Fe(III) is a major source of the iron taken up by phytoplankton in various experimental conditions (Morel et al., 2008). The rate of iron uptake by phytoplankton is directly dependent on Fe^3+^ concentration, rather than the concentration of ferric chelates (Anderson and Morel, 1982). However, phytoplankton must nevertheless make use of the iron bound to strong organic ligands in the ocean, and the mechanisms by which iron is released from these complexes should be further explored. It has been suggested that iron can form a ternary complex with its chelator and the iron transporter (prophetically called phytotransferrin by the authors, see below), leading to direct metal exchange (Anderson and Morel, 1982). It has also been suggested that iron is released from its ligand by photochemical activity (Maldonado et al., 2005), or by reduction through cell surface reductase activity (Shaked et al., 2005) or ${\text{O}}_{2}^{-}$ generation (Kustka et al., 2005). Light has been shown to enhance iron acquisition from ligands, suggesting a potentially important role of photochemistry in the uptake of iron from the organic iron pool in surface waters by oceanic phytoplankton (Maldonado et al., 2005). By contrast, superoxide dismutase has no effect on iron uptake by *Thalassiosira weissflogii*, and ${\text{O}}_{2}^{-}$ production by the cells is, therefore, probably not involved in the uptake process (Kustka et al., 2005). Cell surface ferric reductase activity has been demonstrated in several phytoplankton species, and the importance of this activity for iron uptake by the phytoplankton has been discussed in detail (Maldonado and Price, 2000; Shaked et al., 2005; Kustka et al., 2007; Shaked and Lis, 2012; Lis et al., 2015b), leading to the development of a general kinetic model for iron acquisition by diatoms in which the extracellular reduction of all iron species is a prerequisite for iron acquisition (Shaked et al., 2005). This model is based directly on the mechanism of iron uptake in the model eukaryote *Saccharomyces cerevisiae*: in this yeast, reductive iron uptake involves the prior extracellular dissociative reduction of ferric chelates by the cell surface reductases Fre1p and Fre2p, and the free ferrous ions are then taken up by the high-affinity oxidase-permease complex Fet3p-Ftr1p, which re-oxidizes the iron during its transport (Kosman, 2003; Figure 1). The Fre reductases are flavohemoproteins that transfer electrons from intracellular NADPH to diverse extracellular electron acceptors, including ferric chelates and oxygen (Lesuisse et al., 1996). This reduction step is non-specific and almost all ferric complexes can be reduced (Lesuisse et al., 1996). The next step, permeation, is highly specific and involves the multicopper oxidase Fet3p coupled to the permease Ftr1p. The role of copper in iron uptake underlies the interaction between the metabolism of copper and that of iron, as copper-deficient cells also have impaired high-affinity reductive iron uptake (Kaplan and O’Halloran, 1996). The coupling between a non-specific reduction step and a specific permeation step makes it possible for the cells to use diverse ferric chelates as iron sources, after their reductive dissociation. A similar model of reductive iron uptake has been described in the freshwater alga *Chlamydomonas reinhardtii* (Allen et al., 2007). Genome sequencing has revealed that several phytoplankton species have genes homologous to those encoding Fre1p and Fet3p. This is the case for *T. pseudonana*, which has two highly similar iron permeases (TpFTR1 and TpFTR2) and a divalent metal transporter from the NRAMP superfamily (TpNRAMP) (Armbrust et al., 2004; Kustka et al., 2007), for which an interaction, presumably involving a multicopper oxidase, has been found between copper metabolism and iron uptake (Maldonado et al., 2006). However, a number of phytoplankton species have no gene homologous to *FET3* and/or *FTR1*. In *T. pseudonana*, the ferric reductase transcripts are downregulated by iron, and TpFTR is also regulated by iron status, whereas TpFET3 is not (Kustka et al., 2007). Thus, reductive iron uptake probably occurs, at least in *T. pseudonana*. Additional evidence for reductive iron uptake in phytoplankton is provided by the direct observation of inducible (by iron deprivation) iron reductase activity in these cells (as observed in *T. oceanica*, *T. pseudonana*, *T. weissflogii*, and *Phaeodactylum tricornutum*, for example), and by the observation that the addition of ferrous chelators to the medium inhibits iron uptake (Maldonado and Price, 2000; Shaked et al., 2005).

The Fe’ model is an alternative model of iron uptake by eukaryotic phytoplankton. According to this model, the rate of uptake is dependent on the concentration of unchelated Fe species (Hudson and Morel, 1990, 1993; Sunda and Huntsman, 1995). Iron is acquired via the binding of Fe(III)’ (inorganic soluble ferric species) to a surface ligand, leading to its internalization through transfer across the plasma membrane. Several lines of evidence support this model. Studies of *Pleurochrysis carterae* and *T. weissflogii* have shown that iron binding to the cell surface and uptake into cells are sequential transport steps differing in terms of temperature dependence (Hudson and Morel, 1990). Pulse-chase experiments with excess unlabeled iron showed that iron was taken up directly from the surface without re-entering a solution phase and that surface iron was bound to specific sites on the cell surface and not readily exchanged with chelators. The uptake system was highly specific for iron, displayed the saturation kinetics expected of a facilitated transport system, and this transport system was regulated by the iron nutrition status of the cells (Hudson and Morel, 1990). Another study reported a dependence of iron uptake rate solely on Fe(III)’ concentration, suggesting that the concentration of free ferric ions in equilibrium with ferric chelates might be a determinant factor for iron uptake rate (Anderson and Morel, 1982). The authors of this study named the iron transporter phytotransferrin, and calculated a Fe(III) stability constant for phytotransferrin of *K*_Fe(III)_ = 10^19^ M^–1^, whereas the Fe(II) stability constant was *K*_Fe(II)_ = 10^8.1^ M^–1^. For comparison, animal transferrin has an estimated Fe(III) stability constant of about 10^22^ M^–1^ (Anderson and Morel, 1982). A transferrin-like protein involved in iron transport has been described in the halophilic green alga *Dunaliella salina* (Paz et al., 2007).

Some phytoplankton species have no ferric reductase activity at all, implying that iron must be taken up as Fe(III), possibly via the Fe’ model. This is the case for the alveolate *Chromera velia*, for which non-reductive uptake has been clearly demonstrated (Sutak et al., 2010). This is also the case for the green algae (Prasinophyceae) *Micromonas pusilla* and *Ostreococcus tauri*, and for the coccolithophore *Emiliania huxleyi* (Sutak et al., 2012). For all these species, iron binding at the cell surface has been shown to be a prerequisite for iron uptake, with the rate of iron uptake proportional to the concentration of Fe(III)’ (Sutak et al., 2010, 2012). For species that have a ferric reductase activity, Fe(III)’ (rather than ferric chelates) can also be the main iron source for iron uptake by the reductive pathway: this is the Fe(II)s model in which the cell surface concentration of reduced iron (Fe(II)s) controls uptake. This model includes the Fe’ model by making Fe(III)’ the major source of iron for reductive uptake (Morel et al., 2008). This model of reductive iron uptake should be distinguished from the FeL model (where L is an iron ligand) in which chelated Fe(III) is the direct source of reduced iron in the bulk medium, and both Fe(II)’ and Fe(II)L are the iron sources for uptake (Morel et al., 2008). In the Fe(II)s model (as in the Fe’ model), but not in the FeL model, the iron uptake rate is inversely proportional to the ligand concentration ([L]), which is in accord with some experimental data (Morel et al., 2008). Other experimental data show that the decrease in iron uptake rate resulting from an increase in ligand concentration depends on whether the ferric reductase of the cells is induced or not: the effect of increasing the ligand concentration is much less pronounced when the cell ferric reductase activity is induced (Morrissey et al., 2015), and in this case the FeL model could probably apply. In summary, iron uptake can be reductive or non-reductive (Morrissey et al., 2015), and non-reductive uptake directly depends on the concentration of Fe(III)’ (Fe’ model), while reductive uptake may depend on either the Fe(III)’ concentration (Fe(II)s model) or on the FeL concentration (FeL model). The rate of iron uptake is controlled thermodynamically in non-reductive iron uptake, while a kinetic control applies for reductive iron uptake (Morrissey et al., 2015). Non-reductive uptake by phytotransferrin (see below) is probably more specific and has probably higher affinity for iron than reductive uptake; this mechanism of uptake is likely to be an adaptation to the low iron environment of open ocean (Morrissey et al., 2015). Several studies and genomic data have strongly suggested that no one model, whether reductive or non-reductive, can account for all iron uptake, with several different iron uptake pathways coexisting in a single species (Morrissey and Bowler, 2012; Sutak et al., 2012; Raven, 2013; Morrissey et al., 2015). For example, under iron starvation, *P. tricornutum* first induces the non-reductive iron uptake pathway (within 3–5 days of iron limitation) and induces the ferrireductase activity later (after at least 7 days of iron limitation) (Morrissey et al., 2015).

Several phytoplankton species can take up iron from different siderophores. *P. tricornutum* can take up iron more rapidly from FOB and FOE than from iron bound to EDTA (the source of iron in this case is Fe’; note, however, that the authors did not work on EDTA buffered medium), and its uptake of iron from FOB, but not from FOE, is inhibited by the ferrous chelator bathophenanthroline disulfonate, suggesting the existence of multiple transport mechanisms for iron uptake from exogenous siderophores (Soria-Dengg and Horstmann, 1995). Siderophore uptake by *P. tricornutum* will be reviewed in more detail below. *T. oceanica* can also use FOB as an iron source, probably via a reductive mechanism (Maldonado and Price, 2001). No eukaryotic phytoplankton species has yet been shown to excrete a siderophore (Hopkinson and Morel, 2009), so the use of exogenous bacterial siderophores by micro-algae may constitute a basis for algal-bacterial mutualism, as suggested by the work of Amin et al. (2009) for *Marinobacter* and the dinoflagellate *Scrippsiella trochoidea* for the use of vibrioferrin. However, *Pseudo-nitzschia* can secrete domoic acid, which is structurally similar to the phytosiderophore mugineic acid excreted by plants of the Gramineae family. Domoic acid, which is a neurotoxin, chelates iron and copper (Rue and Bruland, 2001) and may promote iron solubilization from sediments in coastal regions (Rue and Bruland, 2001). The addition of domoic acid to natural cultures promotes iron uptake and the growth of *Pseudo-nitzschia* (Wells et al., 2005). Despite its relatively low affinity for iron, domoic acid may be present at a sufficiently high concentration in naturally occurring blooms to facilitate iron uptake (Rue and Bruland, 2001). But these are just hypotheses since the physiological role of domoic acid is currently not known with certainty (Bates et al., 2018).

### *Phaeodactylum tricornutum* as a Model Organism for Studying Iron Uptake in Eukaryotic Phytoplankton

*Phaeodactylum tricornutum* has emerged as the main diatom model organism, particularly for investigations of iron metabolism. It is adapted to low-iron conditions and can grow at iron levels 50-fold lower than the minimum required by *T. pseudonana* (Kustka et al., 2007), like species native to the HNLC regions of the oceans. A high-quality genome sequence has been published for this species, facilitating transcriptome annotation, and genetic modification by modern biological techniques is possible, to generate gene knockouts and fusion proteins for studies of genes in their native organism (Karas et al., 2015; Nymark et al., 2016).

A transcriptomic analysis of *P. tricornutum* showed that *FRE* genes (*FRE1-FRE4*, ferric reductase genes) were induced by iron starvation. *FRE2* is located immediately adjacent to a highly iron-responsive gene encoding a putative ferrichrome-binding protein (*FBP1*) similar to the FhuD protein of the bacterial ferrichrome transport system (Allen et al., 2008). There is no Fet3p or Ftr1p homolog in *P. tricornutum*, but a gene encoding an Irt-like protein of the ZIP family was also found to be induced by iron limitation, potentially accounting for ferrous iron uptake (Allen et al., 2008). A group of iron-regulated genes encoding proteins of unknown function (ISIPs, or iron starvation-induced proteins) common to other low-iron-quota marine diatoms has been identified (Allen et al., 2008). *ISIP* genes are used as markers of iron limitation, as they are inversely correlated with iron availability, as TARA Oceans data showed (Caputi et al., 2019). ISIP2a has been shown to concentrate Fe(III) at the cell surface (Morrissey et al., 2015). Cell lines carrying antisense RNA directed against *ISIP2a* (*ISIP2a* knockdown lines) had lower levels of ferric iron uptake, but not of ferrous iron uptake, than the wild type, particularly in conditions in which the ferrireductase activity of the cells was not induced, and the heterologous expression of *ISIP2a* increased iron uptake in both *Saccharomyces cerevisiae* and *Escherichia coli* (Morrissey et al., 2015). The cell surface localization of the protein was confirmed in *P. tricornutum* by generating transgenic lines containing the *ISIP2a* gene fused to *YFP*. Therefore, it has been suggested that ISIP2a may play a role in iron binding at the cell membrane, consistently with the fact that purified ISIP2a protein is able to bind ferric iron (Morrissey et al., 2015). These findings indicate that ISIP2a contributes to a thermodynamically controlled iron uptake process, compatible with the Fe’ model, in addition to the reductive, kinetically controlled mechanism of iron uptake (Morrissey et al., 2015). The ecological significance of ISIP2a is evident from its expression in as diverse marine lineages as diatoms, dinoflagellates and haptophytes, as demonstrated in metatranscriptomic datasets from Antarctica and Monterey Bay. Moreover, ISIP2a is distantly related to the Fea1 protein of *C. reinhardtii*, which has been suggested (although never actually demonstrated) to facilitate iron uptake by concentrating iron near the plasma membrane (Narayanan et al., 2011). Another study established that ISIP2a was a phytotransferrin capable of mediating high-affinity ferric iron uptake (McQuaid et al., 2018), 34 years after the existence of phytotransferrin was first postulated (Anderson and Morel, 1982). The reconstruction of phylogenetic histories of ISIP2a and transferrin revealed a common origin among phosphonate-binding periplasmic proteins. Following the disruption of *ISIP2a*, the Δ*ISIP2a* cell line displayed significantly slower growth at low Fe’ concentration, and significantly lower levels of iron uptake from ferric EDTA (Fe’) than the wild type, whereas iron uptake from FOB was not affected (McQuaid et al., 2018). The N-terminal domain of human transferrin fused to the signal peptide and transmembrane anchor of *ISIP2a* and reintroduced into Δ*ISIP2a* fully restored high-affinity iron uptake by *P. tricornutum*, confirming that phytotransferrin is a functional analog of transferrin (McQuaid et al., 2018). Internalization by endocytosis was required for the release of iron bound to phytotransferrin, whereas ${\text{CO}}_{3}^{2-}$ was required for the efficient binding of iron to phytotransferrin and Fe’ uptake rates were positively correlated with ${\text{CO}}_{3}^{2-}$ concentration. Fe’ uptake rates and ${\text{CO}}_{3}^{2-}$ concentration were correlated at environmentally relevant concentrations of ${\text{CO}}_{3}^{2-}$, suggesting that the decrease in ${\text{CO}}_{3}^{2-}$ concentration in seawater due to ocean acidification might decrease phytotransferrin-mediated iron acquisition (McQuaid et al., 2018). This finding is consistent with the observation that the acidification of media containing various iron compounds decreases the rate of iron uptake by diatoms and coccolithophores (Shi et al., 2010).

The uptake of the siderophores FOB and FCH by *P. tricornutum* cells is saturable with a *k*_M_ of about 5–7 nM, and uptake is competitively inhibited by the non-reducible gallium analogs of the siderophores (Ga-DFOB and Ga-DFCH) (Kazamia et al., 2018). In this study, iron uptake was preceded by binding at the cell surface, and was not inhibited by the ferrous iron chelators bathophenanthroline disulfonate and ferrozine; FOB-NBD (FOB coupled to a nitrobenz-2-oxa-1,3-diazole moiety), a fluorescent derivative of FOB, accumulated in intracellular vesicles close to the chloroplast, confirming that siderophore uptake was non-reductive (Kazamia et al., 2018). Cell lines carrying an antisense RNA directed against *ISIP1* (*ISIP1* knockdown lines) displayed no siderophore uptake, and the growth of these cell lines was inhibited in a medium containing FOB as the sole iron source. The vesicular localization of FOB-NBD suggested the role of an endocytosis-based process, and this was confirmed by the use of endocytosis inhibitors. These inhibitors decreased the uptake of FOB and FCH, but not of Fe’ (Fe from ferric EDTA; one should note here that these experiments were performed at high iron concentration (1 μM) with cells with induced ferric reductase activity, a condition at which iron enters the cell by reductive uptake rather than by phytotransferrin-mediated endocytosis (Morrissey et al., 2015). Time-course experiments with fluorescent probes showed that siderophores were delivered to the chloroplast, where iron was released. The *ISIP1* gene is globally abundant and largely diatom-specific. Its precise role in siderophore uptake remains to be determined, but endocytosis was found to be impaired in *ISIP1* knockdown lines, consistent with a role for the product of this gene in this process (Kazamia et al., 2018).

In another study, the adjacent *FRE2* and *FBP1* genes were disrupted (to give the Δ*FRE2* and Δ*FBP1* cell lines) and the FOB uptake was then investigated (Coale et al., 2019). Neither of the knockout lines grew when DFOB was added to the growth medium, and the uptake of FOB and FCH (but not of Fe’) was inhibited in both cell lines. Much less of the gallium analog of FOB (Ga-DFOB) than of FOB was taken up (Coale et al., 2019). The cell surface localization of FBP1 and FRE2 was confirmed by generating transgenic lines containing the *FBP1* and *FRE2* genes fused to mCherry and *YFP*, respectively. FBP1 was also observed in small vesicles close to the plastid (Coale et al., 2019), resembling the vesicles previously shown to contain FOB-NBD (Kazamia et al., 2018). The data obtained therefore seemed to indicate that the siderophores were taken up by a reductive pathway. However, no decrease in the activity of the cell surface ferric reductase was observed in the Δ*FRE2* cell line, and the precise site at which reduction occurs therefore remains unclear, as membrane proteins may be internalized by endocytosis before the reductive step, with a lower pH in intracellular vesicles facilitating the reduction and release of iron from the siderophores.

**FIGURE 2:**
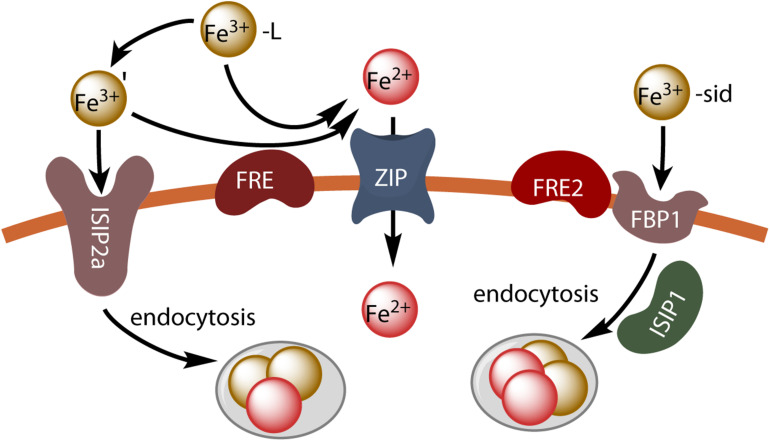
Iron uptake in *P. tricornutum*. Unchelated ferric ions can be transported by endocytosis after binding to the phytotransferrin ISIP2a, or ferric chelates can be dissociated by reduction (FRE) and the resulting ferrous ions can be taken up by divalent metal transporters (ZIP). Hydroxamate siderophores are taken up by endocytosis after binding to the FBP1 protein, and iron is released by reduction (FRE2) possibly in endocytosis vesicles.

The results of these studies suggest that *P. tricornutum* makes use of at least three different inducible iron uptake pathways: a phytotransferrin-mediated non-reductive uptake pathway (Fe’ model), a reductive uptake pathway (reductive model) and a siderophore-mediated uptake pathway. This is illustrated in Figure 2.

### *Ostreococcus tauri* as a Model Organism for Studying Iron Uptake in Eukaryotic Phytoplankton

*Ostreococcus* is a green alga (Prasinophyceae) that has been described as the smallest free-living eukaryote and possesses a very small, dense nuclear genome (about 12.5–13 Mbp). The genomes of two species living in contrasting environments have been sequenced; *O. lucimarinus* lives in oligotrophic waters of the Pacific Ocean (Worden et al., 2004), whereas *O. tauri* lives in eutrophic areas with a high nutrient bioavailability (Botebol et al., 2017). The RCC802 ecotype lives in nutrient-poor environments with a low chlorophyll *a* concentration in surface waters (Botebol et al., 2017). This strain can maintain high growth rates at low iron levels, due to a limited photosynthetic machinery and low protein content, resulting in a lower iron requirement (Botebol et al., 2017). The feasibility of genetic transformation and the existence of vectors for inducible overexpression/knockdown in *O. tauri* have led to the emergence of this species as a model organism for functional genomics and systems biology analyses (Lozano et al., 2014). *O. lucimarinus* has the necessary genes for catecholate (a class of siderophore) biosynthesis, and Palenik et al. (2007) hypothesized that this organism could synthesize its own siderophore, although this seems improbable given that no eukaryotic alga has ever been shown to secrete siderophores (Hopkinson and Morel, 2009). Neither *O. lucimarinus* nor *O. tauri* has genes encoding the multicopper oxidase or permease components of a reductive iron uptake system (Palenik et al., 2007). A transcriptomic analysis of the *O. tauri* cell response to iron limitation provided further insight into the mechanisms involved in iron uptake in this organism (Lelandais et al., 2016). Most of the genes involved in iron uptake and metabolism are regulated by day/night cycles, regardless of iron status (Lelandais et al., 2016). *O. tauri* cells have little or no ferric reductase activity and cannot take up iron from siderophores, but they have been shown to take up Fe^3+^ and Fe^2+^ by an inducible mechanism (Sutak et al., 2012; Lelandais et al., 2016). The cytochrome *b*_561_ proteins (Fre-type proteins) encoded by these cells are probably involved in intracellular iron metabolism rather than extracellular iron reduction. *O. tauri* cells lack the classical components of a reductive iron uptake system, and have no obvious iron regulon. Iron uptake in this species is copper-independent but regulated by zinc (Lelandais et al., 2016). Ferritin, which is involved in the recycling of intracellular iron as a function of day/night cycles, seems to be involved in iron uptake in *O. tauri*, because a Δ*Ftn* mutant (with a disrupted ferritin gene) was found to have impaired iron uptake (Botebol et al., 2015), maybe because iron that can be sensed by the cells is increased in the Δ*Ftn* mutant. Iron uptake may be mediated by a ZIP-family protein (Ot-Irt1) for Fe^2+^ and a new Fea1-related protein (distantly related to phytotransferrin) for Fe^3+^ (Ot-Fea1) (Lelandais et al., 2016). *Ot-IRT1* is strongly induced in the middle of the day after prolonged adaptation to iron deficiency, consistent with the observed peak in the ferrous iron uptake capacity of *O. tauri* at this point in the daily cycle (Botebol et al., 2014). The Ot-Fea1 protein contains several motifs thought to play a key role in iron transport by fungal Ftr1 proteins (Fang and Wang, 2002) – R/K-E/D-X-X-E and R/K-E-X-X-E/D – and it has been shown experimentally to bind iron (Lelandais et al., 2016). Its expression was modulated by Zn, and the protein was purified in association with ferric iron (Scheiber et al., 2019). A phylogeny of homologous Fea1 and Isip2a domains from algal proteins showed this protein to be widespread in different algal groups, with multiplication of the Fea1 domain clearly having occurred on several independent occasions (Lelandais et al., 2016).

## Prokaryotes

Cyanobacteria have significantly higher iron demand than heterotrophic prokaryotes and it was shown that the model cyanobacterium *Synechocystis* 6803 affords the luxury of storing as much as 50% of cellular iron in bacterioferritin (Keren et al., 2004).

### Siderophore-Mediated Iron Uptake

Siderophores represent an important dynamic component of the marine ligand pool. In the eastern Pacific Ocean, siderophore concentrations in iron-deficient regions were estimated to be 9 pM in the form of amphibactins (amphiphilic siderophores with cell membrane affinity) while ferrioxamine siderophores (1–2 pM) were found in coastal regions (Boiteau et al., 2016). Two types of siderophore, FOG and FOE, have been detected in the Atlantic Ocean, at total concentrations of between 3 and 20 pM (Mawji et al., 2008). Siderophores are, thus, an important component of the marine iron cycle, and are a potentially important source of iron for phytoplankton. Diffusive loss of siderophores (Völker and Wolf-Gladrow, 1999) can be avoided when amphiphilic siderophores (many marine siderophores have a non-polar fatty acid tail) are associated with the cell membrane (Hider and Kong, 2010; Arstol and Hohmann-Marriott, 2019).

**FIGURE 3:**
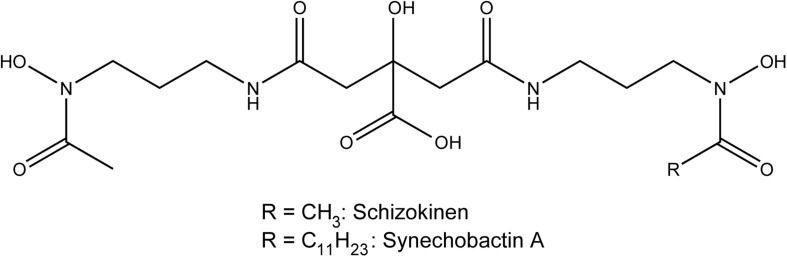
Structure of the siderophores schizokinen and synechobactin A.

Heterotrophic marine bacteria and some cyanobacteria produce large numbers of siderophores, many of which have been isolated and characterized structurally (Arstol and Hohmann-Marriott, 2019; Chen et al., 2019). Siderophores from marine microorganisms can be classified into seven different types on the basis of their functional groups and hydrophobicity: hydroxamates, α-hydroxycarboxylates, catecholates, mixed hy- droxamates/α-hydroxycarboxylates, mixed α-hydroxycarboxy- lates/catecholates, mixed hydroxamates/catecholates and other types of siderophores (Chen et al., 2019). Hydroxamate, catecholate and mixed-type siderophores can be detected in the supernatant of growing cyanobacteria cultures (Wilhelm and Trick, 1994). Synechobactins provide an example of mixed hydroxamates/α-hydroxycarboxylates. They are produced by the marine cyanobacterium *Synechococcu*s sp. PCC7002 (Ito and Butler, 2005), and their structure is presented in Figure 3. Synechobactins are a suite of siderophores derived from schizokinen (produced by *Anabaena* species), which chelates iron via two α-hydroxamate groups and one α-hydroxycarboxylate group. Marine siderophores, unlike terrestrial ones, often show α- or β-hydroxy acid moieties, resulting in photolability of the ferric complex (Barbeau et al., 2001). In some cases, siderophores can bind metals other than iron and are involved in other functions, such as protecting cells against copper toxicity (Clarke et al., 1987).

**FIGURE 4:**
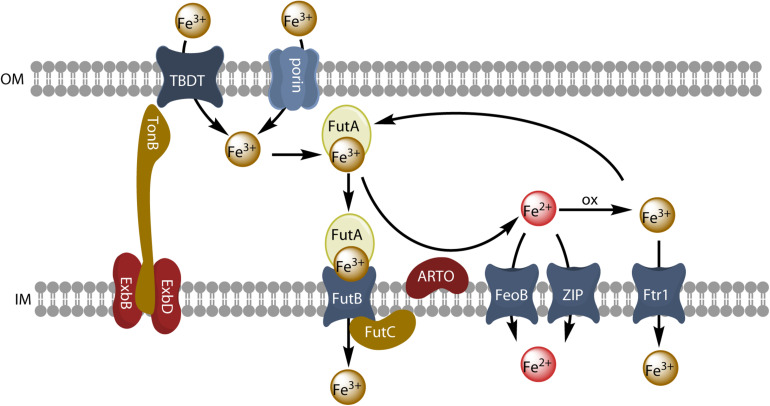
Model of reductive iron uptake and uptake of inorganic ferric species. Inorganic ferric species are transported through the outer membrane by TBDT or by porins, and are taken up by the ABC transporter system FutABC. Alternatively, ferric iron of the periplasmic space can be reduced by the alternative respiratory terminal oxidase (ARTO) and the free ferrous ions can be transported by divalent metal transporters (FeoB or ZIP). After reoxidation, iron can be taken up by the high-affinity permease Ftr1.

The biosynthesis of siderophores by cyanobacteria involves either non-ribosomal peptide synthases (NRPSs), which catalyze the peptide bonds between amino acids of the siderophore backbone, or NRPS-independent synthases (NIS). In some cases, NRPSs are accompanied by polyketide synthases (PKSs), catalyzing the condensation of carboxylate groups (Staunton and Weissman, 2001). Citrate-based siderophores, such as schizokinen and synechobactins, are produced by NIS systems (Arstol and Hohmann-Marriott, 2019). The process of siderophore export by bacteria is not well documented. Three different types of proteins have been implicated in this process in bacteria: the major facilitator superfamily (MFS), the resistance nodulation and cell division (RND) superfamily and the ATP-binding cassette (ABC) superfamily (Arstol and Hohmann-Marriott, 2019). A siderophore export system involving the inner membrane MFS protein SchE has been described in the cyanobacterium *Anabaena* sp. PCC7120 (Nicolaisen et al., 2010). The import of iron-loaded siderophores is largely similar in all bacteria. At the outer membrane, TonB-dependent transporters (TBDTs) mediate the transport of extracellular siderophores into the periplasmic space. These transporters show high affinity and high specificity for siderophores. The structure of a TBDT consists of a 22-stranded β-barrel with a N-terminal globular plug domain within the barrel. This plug domain binds the siderophore and interacts with the Ton system of the inner membrane (Noinaj et al., 2010). This Ton system includes TonB (the energy-transducing element, which protrudes in the periplasmic space) and the integral proteins ExbB and ExbD (the structural elements) (see Figure 4). TonB recognizes a TonB-box in the TBDT and acts as an energy transducer, by coupling the proton-motive force of the cytosolic membrane to the outer membrane transporter (Kranzler et al., 2013). The interaction between TonB and the TBDT loaded with a siderophore induces a conformational change of the plug domain, leading to the siderophore transfer through the channel of the TBDT [reviewed in Kranzler et al. (2013) and Arstol and Hohmann-Marriott (2019)]. Once in the periplasm, the siderophores are first bound to a periplasmic protein and then transported across the inner membrane, often by ABC-transporters (Noinaj et al., 2010). Consistent with their role in iron transport, all TBDTs for siderophores are controlled by the Fur transcriptional repressor, and their expression is, therefore, repressed when iron concentration reaches a certain level (Noinaj et al., 2010). Iron complexes are the principal substrates of TBDTs, but other substrates are also transported by this mechanism (namely, vitamin B_12_, nickel chelates and carbohydrates). Heme, which can be used as an iron source by numerous heterotrophic bacteria (Hopkinson et al., 2008; Hogle et al., 2017), is also transported in this way. TBDTs are widely distributed in heterotrophic bacteria, but are not present in all cyanobacteria, although they are present in various ecotypes of the ecologically important *Prochlorococcus* (Malmstrom et al., 2013) and *Synechococcus* strains (Ahlgren et al., 2020). Despite their abundance in heterotrophic bacteria, siderophores do not appear to be widely produced by marine cyanobacteria (Hopkinson and Morel, 2009). Bioinformatic analyses of the molecular systems involved in siderophore synthesis were performed on freshwater and marine cyanobacteria genomes, and NRPS/PKS and NIS genes were absent from the picocyanobacterial lineages (*Prochlorococcus* and *Synechococcus*) dominating primary production (Ehrenreich et al., 2005; Hopkinson and Morel, 2009). Many species make use of iron from siderophores that they do not produce themselves (xenosiderophores) (Babykin et al., 2018), by mechanisms of uptake that could be Ton B-dependent or Ton B-independent.

### Reductive Iron Uptake and Uptake of Inorganic Ferric Species

Many microorganisms have genes encoding multiple iron transporters. By analyzing prokaryotic genomes and metagenomics, Hopkinson and Barbeau (2012) concluded that Fe^3+^ ABC transporters were the most abundant iron transporters. The Fe^3+^ ABC transporter of the model freshwater cyanobacterium *Synechocystis* PCC6803 consists of three subunits: the FutA2 (ferric uptake transporter A2) protein is a periplasmic substrate-binding protein that associates with its partners, FutB and FutC, both anchored in the inner membrane, to form an active Fe^3+^ uptake ABC transporter, FutABC (Katoh et al., 2001; see Figure 4). In many cases, the addition of an iron-trapping reagent, such as ferrozine or bathophenanthroline disulfonate, inhibits the uptake of iron from an inorganic iron source or siderophores in bacterial cells. This implies that iron must be reduced before uptake, as in the eukaryotic yeast and *Chlamydomonas* models. It has been suggested that this reductive iron uptake strategy operates in many cyanobacteria (Kranzler et al., 2011, 2013, 2014; Lis et al., 2015a; Rudolf et al., 2015; Xu et al., 2016; Eichner et al., 2019). The outer membrane has no obvious redox activity. Iron must therefore probably be reduced either in the periplasm or at the surface of the inner membrane. The alternative respiratory terminal oxidase (ARTO) of the inner membrane has been identified as a possible ferric reductase, as a Δ*ARTO* strain of *Synechocystis* PCC 6803 has decreased ferric reductase activity and decreased iron uptake rate (Kranzler et al., 2014), and other studies have suggested that iron is reduced by superoxide, particularly in the nitrogen-fixing *Trichodesmium erythraeum* (Roe and Barbeau, 2014). ARTO has been shown to be expressed and upregulated in response to lower iron availability in *Trichodesmium* (Polyviou et al., 2017). In some cases, iron may be re-oxidized by oxygen and transported through the inner membrane by an FTR1 (cFTR1) permease similar to that found in yeast (Xu et al., 2016). Ferrous iron would then be transported into the cell by the FeoB protein and/or by divalent metal ion transporters (NRAMP or ZIP transporters for picocyanobacteria) (Hopkinson and Barbeau, 2012). Iron may be transported across the outer membrane to the periplasm by an active mechanism mediated by a TBDT, or a passive diffusion mechanism mediated by porins, which are trimeric outer membrane β-barrel proteins (Qiu et al., 2018). When it reaches the periplasm, ferric iron is bound by FutA2, generating a chemical gradient to facilitate iron influx (Kranzler et al., 2014). Reductive iron uptake is widespread in cyanobacteria (Lis et al., 2015a) and may involve diverse ferric chelates as substrates and various siderophores remaining outside the cell during the uptake process (Kranzler et al., 2013).

Reductive iron uptake has several advantages compared to the siderophore-mediated iron uptake strategy (although both strategies are not mutually exclusive). A great number of ferric chelates can be reduced and dissociated by an unique non-specific reductive system, and the drawback of the use of siderophores is their high diffusion rate in the medium (if the siderophores are not anchored to the cell by a lipophilic tail) (Völker and Wolf-Gladrow, 1999). In addition, siderophore biosynthesis and transport is energetically less favorable. In fact, a comparative study of iron uptake in marine and freshwater cyanobacteria suggests that the main strategy of iron acquisition in these microorganisms is the reductive pathway, alone or in combination with siderophore-mediated pathway (Lis et al., 2015a).

### *Synechocystis* sp. Strain PCC 6803 as a Model Cyanobacterium

Although this species is a freshwater alga, it can be considered as a model cyanobacterium. The mechanisms of iron uptake have been well studied in this organism. Genes encoding components of siderophore synthesis are absent in this species (Hopkinson and Morel, 2009), but it is able to use siderophores produced by other organisms, such as FOB, schizokinen, aerobactin (Kranzler et al., 2011) or hydroxamate siderophores produced by the cyanobacterium *Anabaena variabilis* ATCC 29413 (Babykin et al., 2018; Obando et al., 2018). One TonB protein and four TBDTs are present in *Synechocystis* 6803, and expression of the *TonB* gene and of the TBDT-encoding genes was induced by iron limitation (Qiu et al., 2018). Moreover, a mutant disrupted for the *TonB* gene showed defective growth on low iron medium, and a quadruple mutant in which the four TBDTs were inactivated showed induction of iron uptake genes such as *futA* and *feoB* (Qiu et al., 2018). The quadruple mutant also showed defective iron uptake from both siderophores (FOB) and inorganic ferric species (Fe’), although residual iron uptake was higher with Fe’ as iron source, suggesting that Fe’ could be taken up by another pathway (Qiu et al., 2018). There are six porins expressed at the outer membrane of *Synechocystis* 6803, and it was shown (by generating mutants) that these porins were involved in residual Fe’ uptake (Qiu et al., 2018). Thus, at the outer membrane TBDTs are not only involved in siderophore uptake, but also in the uptake of inorganic ferric species together with porins. At the inner membrane, other studies have shown that ExbB-ExbD complexes are essential for inorganic iron uptake (Jiang et al., 2015), and thus the whole TonB system is involved in the uptake of siderophores and of inorganic iron species. Further steps of ferric iron uptake involve the ABC transporter, FutABC (Katoh et al., 2001) as described above. Alternatively, ferric iron is reduced at the inner membrane and ferrous iron is taken up by the FeoB protein (Kranzler et al., 2014). Iron uptake and homeostasis is regulated by the FurA and PfsR transcriptional regulators (Cheng and He, 2014). A model of iron uptake from inorganic ferric species by cyanobacteria is presented in Figure 4.

### *Trichodesmium* and the Use of Iron From Dust

The globally important diazotroph *Trichodesmium* can use desert dust as a source of iron (Rubin et al., 2011; Polyviou et al., 2017). Atmospheric dust is also a significant source of iron in iron-limited regions of the ocean, but this dust rapidly sinks below the surface and the low solubility of iron from dust restricts its acquisition by phytoplankton. *Trichodesmium* colonies can capture dust particles in their core, and Kessler et al. (2020) have shown that these colonies display a preference for iron-rich particles over iron-free particles. The preferred collection of iron-rich particles and rejection of iron-free particles suggest the ability to sense iron and selectively utilize iron-rich dust particles (Kessler et al., 2020). The ability of a cyanobacterium to solubilize ferrihydrite particles has been shown in *Synechocystis* PCC 6803 (Kranzler et al., 2016). The main mechanisms for dissolving mineral iron from dust include reductive dissolution and siderophore-mediated dissolution, both being thought to be involved in *Trichodesmium* dust utilization (Basu et al., 2019; Eichner et al., 2019). The presence of H_2_ strongly stimulates mineral iron uptake by natural colonies of *Trichodesmium*, and it has been suggested that H_2_ acts as a source of electrons for the reduction of mineral iron by the cells (Eichner et al., 2019). However, *Trichodesmium* colonies host many associated siderophore-producing bacteria (Basu et al., 2019). These siderophores have been shown to solubilize iron from its mineral state in dust and to make it available to *Trichodesmium* cells (Basu et al., 2019). It is not known whether *Trichodesmium* takes up siderophores by a reductive or non-reductive mechanism, but the cells may contain proteins capable of siderophore transport (Polyviou et al., 2018). Interactions of this type are typically mutualistic, with the heterotrophic bacteria facilitating *Trichodesmium* iron acquisition and *Trichodesmium* colonies providing the carbon and nitrogen substrate for bacterial colonization in the form of exudates (Basu et al., 2019). Other examples of mutualism between siderophore-producing bacteria and other phytoplankton species able to use siderophores are likely to be discovered in the future, as suggested for vibrioferrin-producing *Marinobacter* living in the phycosphere of coccolithophores and dinoflagellates (Amin et al., 2009).

## Current Developments and Conclusion

**FIGURE 5:**
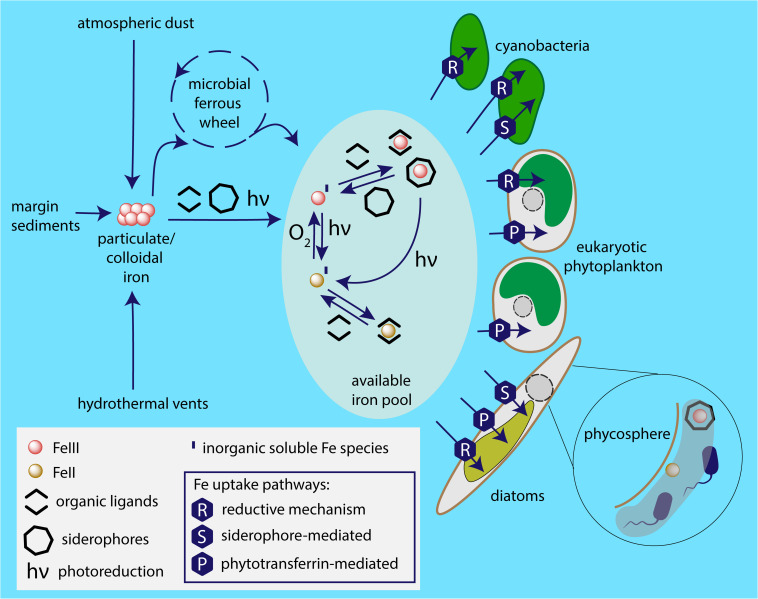
Iron sources available to marine phytoplankton and employed uptake pathways. Particulate iron from sources including atmospheric dust, glaciers, coastal sediments, hydrothermal vents is dissolved by the photochemical reactions, complexation and the microbial ferrous wheel (Kirchman, 1996). The enormous complexity of the mechanisms and species behind the biological iron recycling is only recently being fully acknowledged, involving organisms as diverse as viruses, both heterotrophic and photosynthetic bacteria and protists, mesozooplankton (Boyd and Ellwood, 2010). Different species of iron are available to phytoplankton based on marine chemistry, i.e., ferric and ferrous ions; in inorganic form or chelated to organic ligands. It appears that the main strategy of cyanobacterial iron acquisition is the reductive iron uptake that can be combined with siderophore-mediated pathway. The common iron uptake mechanism employed by eukaryotic phytoplankton is mediated by phytotransferrin, while some species additionally use reductive iron uptake and (at least) some diatoms are able to acquire xenosiderophores. Phycosphere represents a unique environment in the close proximity to algal cell surface, where iron availability may be significantly increased due to bacterial iron metabolism.

In the few last decades, huge progress has been made in our understanding of the fascinating and complicated mechanisms phytoplankton employs to cope with extremely low iron levels in the marine environment. These mechanisms are synthesized in Figure 5. For example, in eukaryotes, a major breakthrough has been the identification and characterization of phytotransferrin (McQuaid et al., 2018). Recent advances in genetic manipulation tools, improved availability and quality of proteomics and transcriptomics technologies, as well as the increasing number of sequenced genomes, shifted the routes of the current investigations and in the near future we can expect to deepen our knowledge of the molecular aspects of microbial iron acquisition in oceans. A key approach toward the complex understanding of iron metabolism in ocean appears to be meta-omics. A remarkable pioneering metatranscriptomic analysis of microcosm iron enrichment by Marchetti et al. (2012) provided a novel insight into the mechanisms behind the ecological success of diatoms in the environment with changing iron availability and highlighted the role of ISIPs in eukaryotic phytoplankton iron homeostasis. Naturally, when discussing the significance of meta-omics in studying any aspects of marine phytoplankton metabolism, the importance of the Tara Ocean expedition has to be emphasized. This unprecedented project revealed an unexpected diversity of the species participating in the biological iron recycling, highlighting, e.g., so far unknown importance of parasitic protists in the marine microbial interactions (de Vargas et al., 2015). Together with currently ongoing international program aimed to systematically study the distributions of key trace elements and isotopes, the GEOTRACES (Anderson, 2020), we can now start to obtain the complex view of the marine biogeochemical cycle of iron. In fact, the first analysis of the Tara Ocean metagenomic and metatranscriptomic data sets in relation to iron availability based on global-scale biogeochemical models has recently been published by Caputi et al. (2019). This study, revealing the complexity of adaptive and acclimatory strategies employed by marine phytoplankton to overcome iron stress, provides a proof of concept that integration of omics datasets with biogeochemical models represents an important part of modern oceanography.

A better understanding of iron uptake mechanisms in different groups of phytoplankton might help us to better predict how these organisms will fare in a changing ocean.

## Author Contributions

All authors listed have made a substantial, direct and intellectual contribution to the work, and approved it for publication.

## Conflict of Interest

The authors declare that the research was conducted in the absence of any commercial or financial relationships that could be construed as a potential conflict of interest.

## Abbreviations

FOB: ferrioxamine B
FOE: ferrioxamine E
FOG: ferrioxamine G
FCH: ferrichrome.
